# Using Gold-Nanorod-Filled Mesoporous Silica Nanobeads for Enhanced Radiotherapy of Oral Squamous Carcinoma

**DOI:** 10.3390/nano11092235

**Published:** 2021-08-30

**Authors:** Mei-Hsiu Chen, Ming-Hong Chen, Chia-Ying Li, Fu-I Tung, San-Yuan Chen, Tse-Ying Liu

**Affiliations:** 1 Department of Internal Medicine, Far Eastern Memorial Hospital, New Taipei 220, Taiwan; michelle8989@gmail.com; 2 Department of Biomedical Engineering, Ming Chuang University, Taoyuan 333, Taiwan; 3 Graduate Institute of Nanomedicine and Medical Engineering, Taipei Medical University, Taipei 110, Taiwan; chen.minghong@gmail.com; 4 Department of Neurosurgery, Wang Fang Hospital, Taipei Medical University, Taipei 116, Taiwan; 5 Department of Biomedical Engineering, National Yang Ming Chiao Tung University, Taipei 112, Taiwan; cccaandy@gmail.com; 6 Department of Orthopaedic Surgery, Taipei City Hospital, Taipei 112, Taiwan; fui.tung@gmail.com; 7 Department of Health and Welfare, College of City Management, University of Taipei, Taipei 112, Taiwan; 8 Department of Materials Science and Engineering, National Yang Ming Chiao Tung University, Hsinchu 300, Taiwan; sanyuanchen@nctu.edu.tw

**Keywords:** radiotherapy, mesoporous silica, gold nanoparticles, radiosensitizer, oral cancer

## Abstract

Radiotherapy (RT), in combination with surgery, is an essential treatment strategy for oral cancer. Although irradiation provides effective control over tumor growth, the surrounding normal tissues are almost inevitably affected. With further understanding of the molecular mechanisms involved in radiation response and recent advances in nanotechnology, using gold nanoparticles as a radiosensitizer provides the preferential sensitization of tumor cells to radiation and minimizes normal tissue damage. Herein, we developed gold nano-sesame-beads (GNSbs), a gold-nanorod-seeded mesoporous silica nanoparticle, as a novel radioenhancer to achieve radiotherapy with a higher therapeutic index. GNSbs in combination with 2 Gy irradiation effectively enhanced the cytotoxic activity CAL-27 cells. The well-designed structure of GNSbs showed preferential cellular uptake by CAL-27 cells at 24 h after incubation. Gold nanorods with high density modified on mesoporous silica nanoparticles resulted in significant reactive oxygen species (ROS) formation after irradiation exposure compared with irradiation alone. Furthermore, GNSbs and irradiation induced more prominent DNA double-strand breaks and G2/M phase arrest in CAL-27 than those in L929. In animal studies, radiotherapy using GNSbs as a radiosensitizer showed significant suppression of tumor growth in an orthotopic model of oral cancer. These results demonstrate that using GNSbs as a radiosensitizer could possess clinical potential for the treatment of oral squamous carcinoma.

## 1. Introduction

More than 500,000 new cases of head and neck cancer are diagnosed worldwide each year. This represents an important global health problem [1]. Head and neck cancer commonly develop in the oral cavity. Squamous cell carcinoma is the most common pathological type in oral cancer [2]. Radiation therapy has a vital role in current treatment strategies for oral squamous cell carcinoma. Major improvements in the treatment of oral squamous cell carcinoma have been accomplished by combining sophisticated surgical techniques and radiotherapy (RT) delivery [3]. However, there are several inevitable complications caused by radiotherapy, such as mucositis, osteoradionecrosis, radiation caries, etc. [4]. According to some studies, oral mucositis can occur in most patients undergoing radiotherapy for oral cancer [5,6]. One treatment modality developed for oral cancer is photodynamic therapy (PDT); however, the clinical use of PDT is hindered by the limited tissue penetration of excitation light. To control this problem in conventional PDT, a series of deep PDT techniques using different excitation sources, such as near-infrared (NIR) light, X-ray radiation, or internal self-luminescence, have been explored [7]. Based on the concept of X-ray-mediated PDT, supplementing conventional radiation therapy with photosensitizers will enable the use of lower doses of radiation. Therefore, a radiosensitizer designed to increase the RT energy deposit inside tumor cells to increase tumor cell death when compared with conventional RT alone, without toxicity towards the adjacent healthy tissues, may decrease the possibility of developing the above-mentioned complications [8].

**Figure 1:**
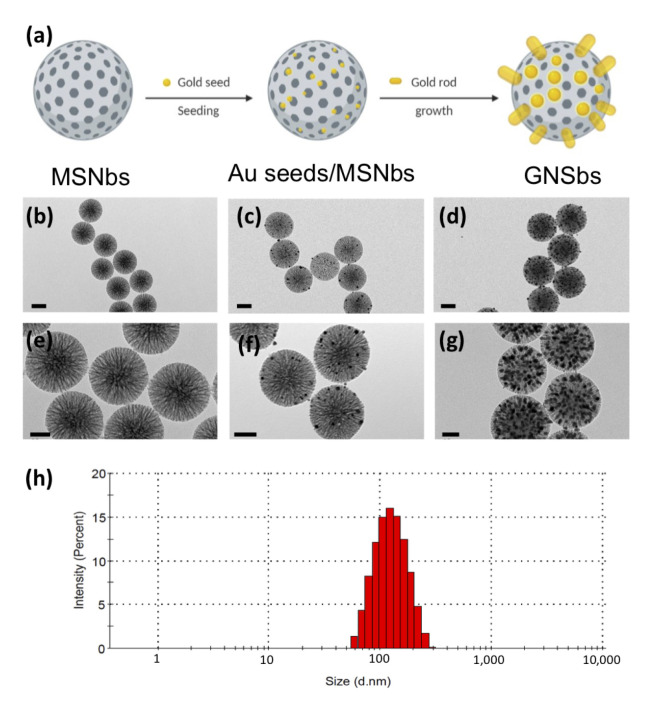
The synthesis and morphology of the gold nano-sesame-beads (GNSbs). (**a**) Schematic illustration of GNSbs. TEM images of (**b**,**e**) the mesoporous silica nanobeads (MSNbs), (**c**,**f**) the gold-seed-filled porous silica nanobeads (GSNbs), (**d**,**e**) the gold nano-sesame-beads (GNSbs). (**h**) The particle size distribution of GNSbs. Scale bar = 100 nm in (**b**,**c**,**d**). Scale bar = 50 nm in (**e**,**f**,**g**).

Introducing high-atomic-number metal into the target tissue during radiotherapy is a feasible approach to maximize the differential effects between tumor and normal tissue response. This concept has led to research on gold-based nanoparticles as a radiosensitizer to improve radiation therapy. Gold nanoparticles (AuNPs) have been extensively studied due to their high X-ray absorption coefficient. In addition, the ease of synthetic manipulation and precise control over the particle’s physicochemical properties make AuNPs a good candidate for radiosensitization [9]. Nonetheless, AuNPs may aggregate in cell culture media, blood, or tissue fluid after exposure to ions and proteins, which may cause unexpected results [10,11,12,13,14,15]. In this sense, silica can bring additional advantages. With a SiO${}_{2}$ coating over the gold nanoparticles, the cytotoxicity and non-specific interactions should be reduced and biological media stability should be improved [16]. However, as a radiosensitizer, simple coatings for gold nanoparticles can result in a decrease in enhancement [17]. Therefore, in this work, we designed a gold nano-sesame-bead (GNSb) by creating mesoporous silica nanobeads (MSNbs) followed by seeding multi-gold nanorods into the pores of silica nanoparticles (Figure 1a). The structure of GNSbs was expected to avoid the undesired aggregation and agglomeration of gold nanorods in the medium. The size of silica nanoparticles could be controlled and allow tumor-specific delivery by utilizing the enhanced permeability and retention effect, without early elimination of nanoparticles from the kidney. On the other hand, the relatively even distribution of nanorods on the silica nanobeads could provide more efficient physical (photoelectrons, Auger electrons, and lower energy secondary electrons) and chemical (radical formation, chemical sensitization of DNA) enhancement mechanisms for radiosensitization.

We aimed to systematically investigate the efficacy of GNSbs in sensitizing oral squamous carcinoma to radiotherapy. We investigated the physical and chemical characteristics of GNSbs with transmission and scanning electron microscopy, energy-dispersive X-ray spectrometry (EDS), X-ray diffractometry (XRD), and Fourier transform infrared spectroscopy (FTIR). The effects and mechanisms of the radiosensitization of GNSbs were evaluated in vitro by cellular uptake, production of reactive oxygen species (ROS), DNA double-strand breaks, and cell cycle analysis using L929 and CAL-27 cells. Furthermore, the therapeutic effects of combining GNSbs and irradiation were analyzed using an orthotopic oral squamous carcinoma model in mice.

## 2. Materials and Methods

### 2.1. Synthesis and Characterization of MSNbs, GSNbs, and GNSbs

#### 2.1.1. Synthesis of Mesoporous Silica Nanobeads (MSNbs)

MSNbs were synthesized using the following procedures. First, 300 mg of cetyltrimethylammonium bromide (CTAB) was introduced into a 70 ${}^{\circ }$C solution containing 45 mL of octane and 140 mL of dilute water. The resulting mixture was magnetically stirred for 20 min. Then, 8.5 mL of styrene monomer, 66 mg of lysine, 3000 mg of tetraethylorthosilicate (TEOS), and 115 mg of 2,2′-azobis(2-amidinopropate) dihydrochloride (AIBA) were magnetically stirred into the solution for 4 h. Afterwards, the suspension was cooled down to room temperature and subjected to centrifugation for 10 min at 6000 rpm. The MSNb products were collected by washing 3 times with pure methanol and removing the template through a 600 ${}^{\circ }$C heat treatment.

#### 2.1.2. Synthesis of Gold-Seed-Filled Porous Silica Nanobeads (GSNbs)

Ice-cold NaBH${}_{4}$ (0.60 mL, 0.010 M) was added to a mixture of CTAB (5 mL, 0.20 M) and HAuCL${}_{4}$ (5 mL, $5\times 10^{-3}$ M). After 2 h of stirring, this seed solution was maintained at a temperature of 25 ${}^{\circ }$C. Then, 1 mM porous silica beads and 1 mL of the seed solution were combined and magnetically stirred for 2 h. The GSNbs were extracted through 10 min of centrifugation at 10,000 rpm and redispersion into 1 mL of dilute water.

#### 2.1.3. Synthesis of Gold Nano-Sesame-Beads (GNSbs)

CTAB (5 mL, 0.10 M) and AgNO${}_{3}$ (0.10 mL, 0.01 M) were combined at a temperature of 25 ${}^{\circ }$C. HAuCl${}_{4}$ (0.5 mL, 0.01 M) and ascorbic acid (55 µL, 0.1 M) were then added to the mixture. The solution after this step was colorless because the ascorbic acid served as a reducing agent. At 27–30 ${}^{\circ }$C, 20 µL of the GSNb solution was added to the solution and it was magnetically stirred for 24 h. The final GNSb products were obtained through centrifugation for a duration of 10 min at 10,000 rpm.

### 2.2. Characterization of MSNbs, GSNbs, and GNSbs

The morphologies and sizes of MSNbs, GSNbs, and GNSbs were observed using surface (JEOL, JSM-7600F, Tokyo, Japan) and transmission electron microscopy (JEOL, JEM-752000EX II, Tokyo, Japan). Particle size and Zeta potential were measured via the dynamic light scattering method (DLS; Malvern Zetasizer Nano Series, ZS90, Worcestershire, UK). The MSNBs’ and GNSBs’ chemical structures were studied using Fourier transform infrared spectroscopy (Spectrum 100FT-IR Spectrometers, PerkinElmer, Waltham, MA, USA). The powder X-ray diffraction (XRD) patterns were extracted with an X-ray diffractometer (XRD, Rigaku D/max 2500 XRD, Tokyo, Japan) with Cu-kα radiation and λ = 1.54178 Å.

### 2.3. Cell Culture

The murine L929 fibroblast cell line was cultured in minimum essential medium (MEM), and the CAL-27 human squamous cell carcinoma was cultured in Dulbecco’s modified Eagle medium (DMEM) in a 5% CO${}_{2}$ incubator at 37 ${}^{\circ }$C. Then, 10% fetal bovine serum (FBS) and 1% penicillin–streptomycin (PS) were added to the culture medium. Renewal of medium was carried out every 2–3 days and cell passage was performed at 80% confluence of cells.

### 2.4. Cell Viability

The L929 and CAL-27 cells were cultured in 24-well plates at a density of 5 × 10${}^{4}$ cells per well containing different concentrations of GNSbs (0, 50, 75, 100, 200, 400 µg mL${}^{-1}$) for 24 h. Cells were washed twice with PBS after removal of media and incubated with 5% Presto Blue reagent for 30 min. The resulting solution was then collected and analyzed at 560 nm excitation and 590 nm emission using a multimode microplate reader (Infinite^®^ 200 PRO, TECAN). To evaluate the therapeutic efficacy of combining GNSbs and radiotherapy, cell viability was evaluated at 24 h following the treatment of GNSbs of various concentrations (25, 50, 75, or 100 µg mL${}^{-1}$), irradiation (100 rad min${}^{-1}$, 2 Gy), or GNSbs plus irradiation.

### 2.5. Cellular Uptake

The L929 and CAL-27 cells were incubated in 12-well plates at a density of 1 × 10${}^{5}$ cells per well with GNSbs (50 μg mL ${}^{-1}$) for 3, 6, 24, and 48 h. After being washed with PBS three times, the cells were harvested and then suspended in Milli Q^®^ water (1 mL). The uptake of GNSbs in the L929 and CAL-27 cells was measured by detecting the gold concentration with inductively coupled plasma mass spectrometry (ICP-MS).

### 2.6. Intracellular ROS Detection Using Flow Cytometry

L929 and CAL-27 cells were seeded in 6-well plates at a density of 3 × 10${}^{5}$ cells per well with or without GNSbs. After 24 h, cells were washed twice with PBS, trypsinized, and stained with CellROX^®^ Deep Red. After treatment with X-ray (6 MeV, a dose rate of 10 rad/min, 2 Gy, Clinac 2100C linear accelerator), the cells were then detected by a flow cytometer (Beckman Coulter’s CytoFLEX, Brea, CA, USA) to evaluate the cellular production of ROS.

### 2.7. DNA Double-Strand Break Monitoring

CAL-27 cells were seeded on confocal plates at a density of 2 × 10${}^{5}$ cells per well with or without GNSbs (75 μg mL ${}^{-1}$). After culture for 24 h, cells were washed twice with PBS and treated with X-ray (6 MeV, a dose rate of 10 rad/min, 2 Gy, Clinac 2100C linear accelerator). After 30 min, cells were fixed with 4% formaldehyde and then permeabilized with Triton X-100. Samples were incubated with primary antibody γH2AX (1:1000) at 4 ${}^{\circ }C$ overnight. Then, fluorescein isothiocyanate (FITC)-conjugated secondary antibody was added after several rounds of PBS washing. Double-strand breaks of DNA were then evaluated using fluorescence microscopy.

### 2.8. Cell Cycle Analysis

CAL-27 and L929 cells were seeded onto 6-well plates at a density of 4 × 10${}^{5}$ cells per well. In the treatment goup, GNSbs (75 μg mL${}^{-1}$) were then added and irradiation (10 rad/min, 2 Gy) was performed. After culture for 24, 48, 72 h, cells were collected and fixed with 70% ice-cold ethanol at −20 ${}^{\circ }C$. Cells were washed with PBS after fixation and then labeled with propidium iodide (20 μg mL${}^{-1}$) in the presence of RNase A (0.2 mg mL${}^{-1}$). Incubation for 30 min was carried out at room temperature in the dark. The cell samples were then analyzed with flow cytometry (Beckman Coulter, Fullerton, CA, USA).

### 2.9. In Vivo Analysis

#### 2.9.1. Animal Models

Six- to eight-week-old female nude mice were purchased from the BioLASCO Taiwan Co., Ltd., Taipei, Taiwan. All animal procedures were performed following the Institutional Animal Care and Use Committee (IACUC) of National Yang-Ming University (NYMU) and approved by IACUC of NYMU. Freshly trypsinized CAL-27 cells (1 × 10${}^{6}$) in PBS (30 μL) were injected into the left mouth wall of each mouse for the induction of orthotopic models of oral squamous carcinoma.

#### 2.9.2. Evaluation of Therapeutic Efficacy of GNSbs

Luciferase-expressing CAL-27 was orthotopically inoculated into the oral wall of mice. Treatment was given to determine the therapeutic efficacy of GNSbs in the CAL-27 mouse model. Then, 24 h after cell injection, the mice (n = 3–4 in each group) were treated with saline (control), GNSbs (in situ injection, 75 μg mL${}^{-1}$), irradiation (2 Gy), or GNSbs (in situ injection, 75 μg mL${}^{-1}$) with irradiation (2 Gy). Treatment was delivered every 3 days for a total dose of 10 Gy (irradiation × 5 times) and 54 mg kg${}^{-1}$ GNSbs (if given). In addition, the oral cancer progression was monitored weekly (day 0, 7, 14, 21) using an IVIS Spectrum imaging system (IVIS Xenogen, Alameda, CA, USA).

### 2.10. Statistical Analysis

We performed all in vitro analyses a minimum of three times. Data are expressed as mean ± SD unless otherwise noted. Comparison between groups was performed using ANOVA and multiple comparisons. *p*-value more than 0.05 means not significant; less than 0.01 is indicated by two asterisks (**); less than 0.001 is indicated by three asterisks (***).

## 3. Results

### 3.1. The Synthesis and Morphology of the Gold Nano-Sesame-Beads (GNSbs)

In this work, we designed and fabricated gold nano-sesame-beads (GNSbs) by first creating mesoporous silica nanobeads (MSNbs) followed by seeding multi-gold-nanorods (GSNbs) (as schematically illustrated in Figure 1a). The mesoporous silica nanobeads were first synthesized using an organic template method. The morphology and particle size of the resulting nanoparticles were analyzed using transmission electron microscopy (TEM). The results are demonstrated in Figure 1b,e. The monodispersed mesoporous silica nanobeads had an average size of 140 ± 20 nm with homogenously distributed columnar pores of 7 ± 2 nm. Subsequent seeding and growth of gold nanorods from the nanopores on MSNbs were then accomplished by immersing the mesoprous silica nanobeads (MSNbs) in to the AuCl${}_{4}^{-}$ solution. Gold nanorods of approximately 3 ± 2 nm in size were found within the columnar porosities of MSNbs (Figure 1c,f). Subsequently, evenly distributed gold nanorods in mesoporous silica were synthesized (Figure 1d,g) in situ by this seed-mediated growth process in the channels of silica nanobeads, which confines the diameter of gold nanorods to a hard silica template. The particle size distribution of GNSbs is demonstrated in Figure 1h. Zeta potential was −23 mV.

### 3.2. Physical Analysis of Gold Nano-Sesame-Beads (GNSbs)

**Figure 2:**
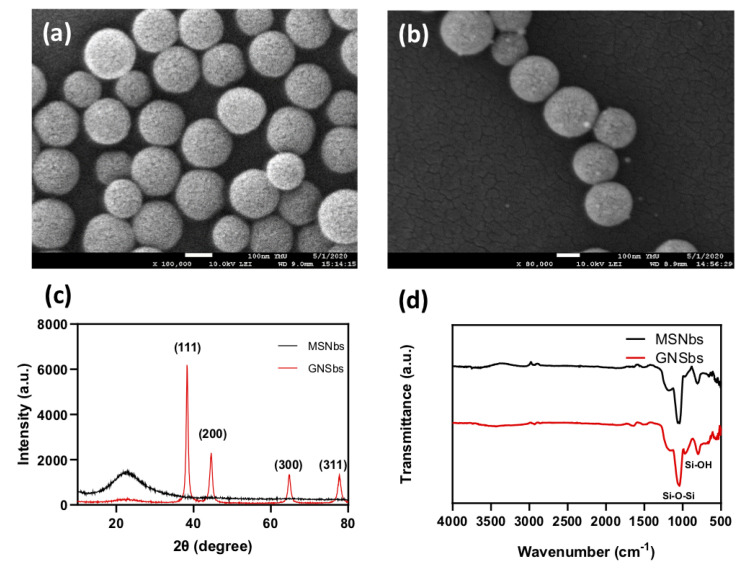
Physical analysis of gold nano-sesame-beads (GNSbs). The scanning electron microscopy analysis of (**a**) the mesoporous silica nanobeads (MSNbs) and (**b**) the gold nano-seasame-beads (GNSbs). (**c**) Typical X-ray diffraction ( XRD ) pattern and (**d**) the FTIR spectra of MSNbs and GNSbs.

The surface morphology and composition of MSNbs and GNSbs were analyzed by surface electron microscopy, as shown in Figure 2. It was observed that both MSNbs and GNsbs showed relatively monodispersed and homogenously distributed pores on the surface, which was consistent with the TEM images. In Figure 2b, as the gold grew and filled up the nanocavities of the silica matrix, gold nanorods evenly protruding from the surfaces of GNSbs were presented. The XRD analysis was carried out to investigate the structure of GNSbs. As shown in Figure 2c, MSNbs exhibited characteristics of non-crystalline silica with a peak at 22.9${}^{\circ }$ [18]. On the other hand, GNSbs showed diffraction peaks at 38.3${}^{\circ }$, 44.5${}^{\circ }$, 64.8${}^{\circ }$, and 77.6${}^{\circ }$, which correspond to the (111), (200), and (220) crystal planes of cubic Au nanoparticles (JCPDS No. 65-2870). The results confirmed the presence of gold nanoparticles. Figure 2d shows the FT-IR spectrum of the MSNbs and GNSbs. The absorption peak at 1020–1110 cm${}^{-1}$ is assigned to the Si-O-Si asymmetric stretching vibration, and the peaks at 960 cm${}^{-1}$ are due to the asymmetric bending and stretching vibration of SiOH. This implied the presence of SiO${}_{2}$ in both MSNbs and GNSbs.

### 3.3. Effect of GNSbs on L929 and CAL-27 Cell Viability and Cellular Uptake

**Figure 3:**
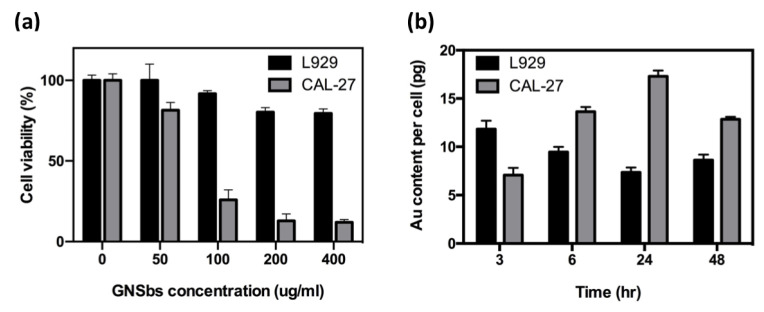
(**a**) Effect of GNSbs on L929 and CAL-27 cell viability after 24 h of treatment. L929 and CAL-27 were treated with various concentrations of GNSbs (0, 50, 100, 200, and 400 μg/mL) for 24 h. (**b**) Cellular uptake analysis by ICP-MS. Cells were exposed to GNSbs at the concentration of 50 μg/mL for 0, 3, 6, 24, and 48 h. Intracellular gold content was analyzed in both CAL-27 and L929 cells. Data were reported as gold content per cell.

To assess the cytotoxicity, L929 and CAL-27 cells were incubated with various concentrations of GNSbs for 24 h. Figure 3a shows the viability of L929 and CAL-27 cells treated with GNSbs at various concentrations. Compared with L929, GNSbs induced a significant decrease in the proliferation activity of CAL-27 cells in a concentration-dependent manner (50, 100, 200, and 400 μg mL${}^{-1}$) after 24 h of culture. High concentrations (200 and 400 μg mL${}^{-1}$) of GNSbs substantially increased the viability (80%) in L929 cells. These results demonstrate that GNSbs induced selective toxicity toward CAL-27 oral cancer cells. Because GNSbs were nontoxic to L929 cells and reduced 27% of the viability of CAL-27 at the concentration of 50 μg mL${}^{-1}$, this dose was chosen for the following cellular uptake and cell cycle analysis and to avoid any adverse effects on L929 cells.

The uptake of GNSbs in the L929 and CAL-27 cells was measured by detecting the gold concentration with inductively coupled plasma mass spectrometry (ICP-MS) [19]. Uptake was detected in both L929 and CAL-27 cells. However, the cellular uptake showed cell-line-specific variability, with the highest level of GNSb uptake at 3 and 24 h for L929 and CAL-27, respectively (Figure 3b). Due to these findings, we decided that radiation treatment should be given 24 h after the administration of GNSbs for oral cancer cells in the following studies.

### 3.4. The Cytotoxicity on CAL-27 and L929 Cells Treated with or without the GNSbs under Radiation

**Figure 4:**
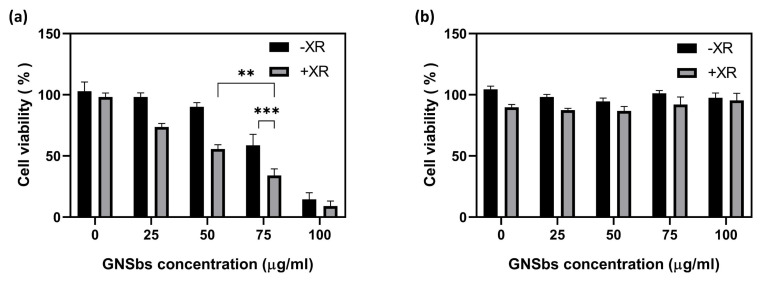
The cytotoxicity of (**a**) CAL-27 and (**b**) L929 cells treated with or without the GNSbs under radiation. The cytotoxicity of CAL-27 cells treated with or without the GNSbs (0, 25, 50, 75, and 100 μg mL${}^{-1}$) under radiation (0 and 2 Gy) at 48 h (*n* = 3, ** *p* < 0.01 and *** *p* < 0.001).

To evaluate the therapeutic efficacy of the combination of GNSbs and radiotherapy, cell viability was evaluated 24 h after treatment with GNSbs at various concentrations, irradiation, or both. As illustrated in Figure 4a, treatment with GNSbs effectively decreased cell viability in a dose-dependent manner. Meanwhile, compared with GNSbs only, treatment with GNSbs and irradiation further decreased cell viability. Furthermore, the reduction in cell viability caused by the combined treatment with GNSbs and irradiation reached the greatest significance when the concentration of GNSbs was 75 μg mL${}^{-1}$. In contrast, L929 cells tolerated the combination of irradiation and various concentrations of GNSbs and retained more than 90% viability (Figure 4b). These results indicate that combining GNSbs and 2 Gy radiation effectively enhanced the therapeutic efficacy in CAL-27 but not in L929 cells. Importantly, the results demonstrate that GNSbs and irradiation had synergistic cytotoxic effects toward CAL-27 cells. The combination of GNSbs and irradiation holds promise as a potential clinical therapy for oral squamous cell carcinoma.

### 3.5. The Intracellular ROS Levels in CAL27 and L929 Cells Treated with or without GNSbs under Radiation

**Figure 5:**
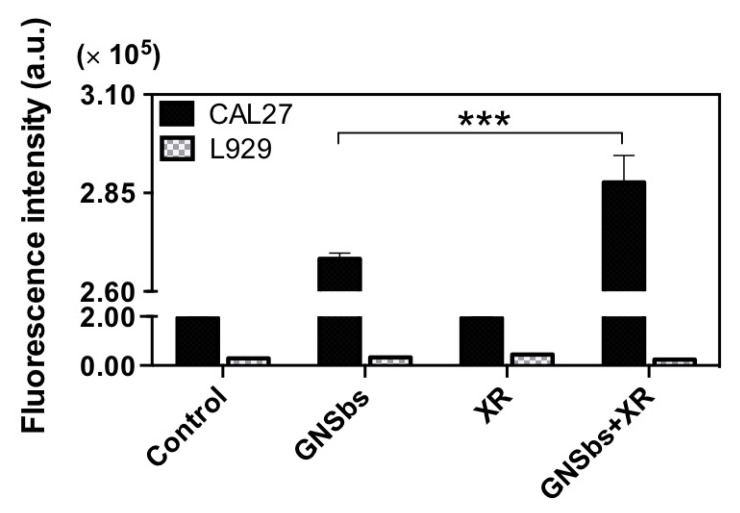
The intracellular ROS levels in CAL27 and L929 cells treated with or without GNSbs (75 μg mL${}^{-1}$) under radiation (0 and 2 Gy). Cells were exposed to GNSbs at the concentration of 75 μg mL${}^{-1}$ for 24 h. Three asterisks (***): *p*-value < 0.001.

Radiolysis of water with subsequent generation of free radicals and ROS is the primary pathway of cell damage induced by radiation [20]. Therefore, we monitored the ROS production in L929 and CAL-27 cells treated with GNSbs, irradiation, or GNSbs plus irradiation. The results shown in Figure 5 suggest that the combination of GNSbs and irradiation could increase intracellular ROS production in CAL-27 specifically. Although CAL-27 demonstrated higher ROS production at the baseline, a significant increase in intracellular ROS was noted after treating CAL-27 cells with irradiation. Importantly, with the addition of GNSbs, the ROS level was substantially further increased in CAL-27 cells. On the contrary, L929 cells maintained a stable ROS level after GNSb, irradiation, or GNSb plus irradiation treatment.

### 3.6. DNA Damage in CAL-27 and L929 Cells Treated with or without GNSbs under Radiation

**Figure 6:**
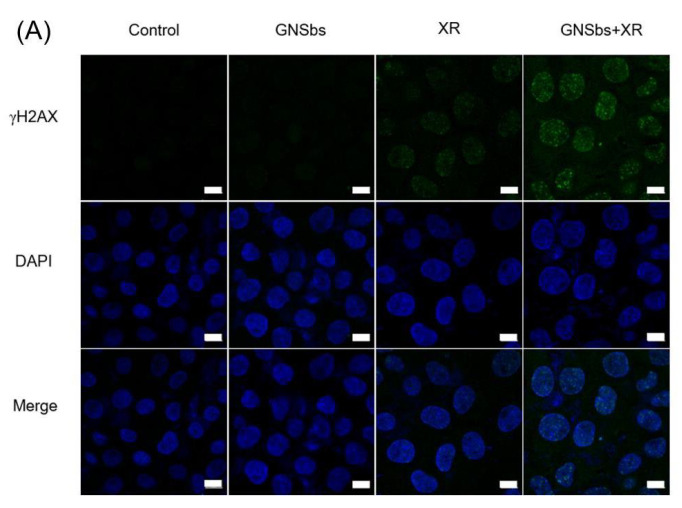

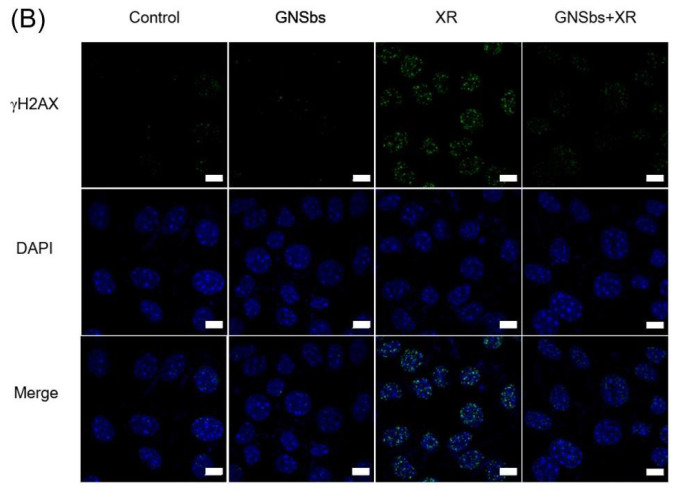
The fluorescence images of DNA damage in (**A**) CAL-27 and (**B**) L929 cells treated with or without GNSbs (75 μg mL${}^{-1}$) under radiation (0 and 2 Gy). The blue and green fluorescence signals represent cell nucleus (DAPI) and DNA double-strand breaks (γ-H2AX), respectively. Scale bar = 10 nm.

Double-strand breaks (DSBs) of DNA are the most detrimental lesions correlated with clonogenic cell death upon radiation exposure [21]. In this study, DNA DSBs were analyzed using the γ-H2AX foci assay. The γ-H2AX foci assay can detect the early events that occur upon DSB formation [21] and identify DNA repair inhibition. This information can provide a possible biological mechanism of radiosensitization for GNSbs. The cells (L929 and CAL-27) were cultured with GNSbs for 24 h before radiation treatment. The results (Figure 6) showed that irradiation caused more DNA damage both in L929 and CAL-27 cells compared to those cells treated without irradiation. In addition, in comparison to irradiation alone, the radiosensitization effect of GNSbs could be noted by the more prominent DNA DSBs in CAL-27 cells. Interestingly, irradiation alone induced more significant DNA damage than GNSbs plus irradiation in L929 cells.

### 3.7. Cell Cycle Analysis of CAL-27 and L929 Treated with Irradiation and GNSbs

**Figure 7:**
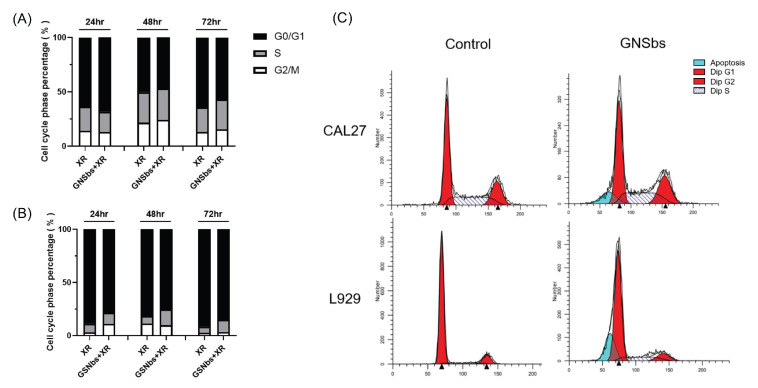
Cell cycle of CAL-27 and L929 treated with 2 Gy XR and GNSbs. The cell cycle phase of (**A**) CAL-27 and (**B**) L929 cells. Cells were treated with or without 75 μg mL${}^{-1}$ GNSbs under radiation (2 Gy) for 24, 48, and 72 h. (**C**) The cell cycle histogram of CAL-27 and L929 cells treated with or without GNSbs (75 μg mL${}^{-1}$) under radiation (2 Gy) at 48 h.

Multiple pathways are involved in the repair mechanisms for double-strand DNA damage of a cell after its exposure to ionizing radiation. However, cell cycle regulation perhaps plays the most crucial role in the sensitivity of cells to ionizing radiation. Cells are most radiosensitive in the G(2)-M phase and radioresistant during the late S phase [22,23]. Therefore, we evaluated whether the combination of GNSbs and radiotherapy worked better by synchronizing cells in the most radiosensitive phase. Different time points after the application of GNSbs and radiotherapy were evaluated to elucidate the optimal temporal treatment strategies for serial treatments in the following animal studies. As shown in Figure 7A,B, more CAL-27 cells remained in the G2/M phase than L929 at each time point (24, 48, and 72 h). At 24 h after combination treatment with GNSbs and irradiation, compared to irradiation alone, there was a significant increase in G2/M phase cells for L929 (Figure 7B). Thus, we believe that the following radiation treatment could be administered at least 48 h after initial GNSb/irradiation combination to avoid injuries to the normal cells. In contrast, the combination of GNSbs and irradiation caused more G2/M arrest in CAL-27 than in L929 cells at 48 h. Furthermore, combining GNSbs with irradiation had more CAL-27 remaining in the G2/M phase than irradiation alone (Figure 7A). As the cell cycle histogram at 48 h after treatment showed that GNSbs with irradiation induced more G2/M phase arrest in CAL-27 than L929 (Figure 7C), it further supported that the duration between each episode of GNSb/irradiation treatment could be 48 h.

### 3.8. Therapeutic Efficacy of GNSbs and Irradiation in the CAL-27 Mouse Model

**Figure 8:**
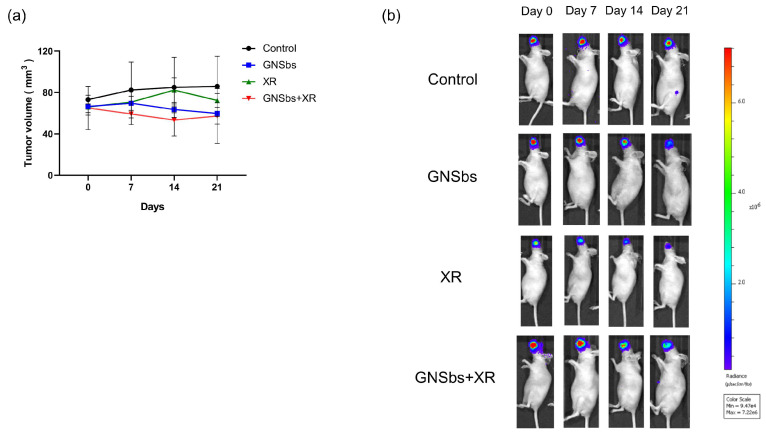
(**a**) The volume of tumors was recorded at day 0, day 7, day 14, and day 21. The treatment started at day 1. (**b**) IVIS images of tumor-induced nude mice treated with GNSbs and X-ray. Each mouse was measured by IVIS on days 0, 7, 14, and 21 after different treatments.

We administered different treatments to the mice with CAL-27 orthotopically injected into the oral wall to evaluate the therapeutic effects of GNSbs. Treatment was delivered every 3 days for a total dose of 10 Gy (irradiation × 5 times) and 54 mg kg^−1^ GNSbs (if given). The volume of the oral cancer was monitored weekly in animals under various treatments. The results are shown in Figure 8. The initial luciferase signal exhibited in individual animals varied. This indicated that the initial size of induced oral cancers can be different due to individual variations in each animal. Despite the difference in the initial size, luciferase expression decreased in the irradiation, GNSb, and GNSb plus irradiation groups (Figure 8b). The GNSb plus irradiation group showed a more significant decrease in the luciferase signal. Figure 8a shows the change in the volume of tumors under various treatments. GNSbs plus irradiation demonstrated a gradual decrease in average tumor size. In the irradiation alone group, the average size of tumor remained stable after 21 days. On the other hand, GNSbs alone showed a smaller decrease in average tumor size. However, the changes in tumor size among each group did not reach statistical significance.

## 4. Discussion

A mesoporous material is a material containing pores with diameters between 2 and 50 nm. Mesoporous silica particles can be synthesized using a simple sol–gel method. In this study, the hydrolysis and condensation reactions of tetraethyl orthosilicate (TEOS) took place in the presence of L-lysine. L-lysine then promoted the formation of silica, resulting in the preparation of the well-ordered silica nanobeads. Lysine was utilized to control silica formation due to its ability to cover the prepared silica after the reaction [24]. On the other hand, AIBA was used as a starter for styrene polymerization. The ability to control the pore size of silica nanobeads could be drastically altered by the styrene concentration [25]. The reactions took place in the system using octane as the hydrophobic-supporting reaction component and cetyltrimethylammonium bromide (CTAB) as the surfactant. Finally, calcination helped to remove the organic components (CTAB and polystyrene) and yield the mesoporous silica nanobeads (Figure 1b,e).

There are several synthetic methods for the preparation of metallic nanorods, such as electrochemical deposition [26] in rigid templates [27], photochemical synthesis [28], and seed-mediated growth [29]. On the other hand, gold species, including AuCl${}_{4}^{-}$ and metallic gold, have a high affinity for amino groups due to electrostatic and/or coordinate interactions. Hence, using amino groups to selectively modify the inner surface of the silica nanobeads allows the formation and growth of gold species inside the templates during the adsorption of gold seeds [30]. Therefore, in this work, we adapted the seed-mediated growth procedure to grow gold nanorods directly on the mesoporous silica nanobeads by using seeds immobilized onto the columnar pores (Figure 1c,f).

After successfully synthesizing gold-seed-filled porous silica nanobeads (GSNbs), they were added into the gold growth solution, wherein gold developed a nanorod geometry (Figure 1d,g) and filled up the columnar pores of MSNbs. It is crucial to keep a low reaction rate and minimize self-nucleation during the growth step of gold seeds [30]. Therefore, we used ascorbic acid as a weaker reducing agent for the reduction of gold salt.

Previously, Paro et al. reported that both analytical and Monte Carlo simulations predicted an increase in the dose enhancement factor with an increasing concentration of nanoparticles [31]. In addition, Leung et al. suggested that the farther from the nanoparticle, the lower counts of secondary electrons emitted from irradiated gold nanoparticles [32]. Therefore, it is reasonable to believe that, in GNSbs, a confined volume with nano-scaled size containing a high concentration of gold is effective for dose enhancement. Furthermore, Leung et al. also reported that there is a tradeoff in designing the sizes of nanoparticles for dose enhancement because the numbers of secondary electrons emitted from nanoparticles and the self-absorption of energy by nanoparticles increased with increasing particle size [32]. Hence, GNSbs, a gold-nanorod-containing structure, could be employed to increase the number of secondary electrons (i.e., long axis) with limited self-absorption of energy (i.e., short axis).

Several pieces of research showed promising selective cytotoxic treatment of cancer cells and targeted therapy by nanoparticles that do not harm normal cells [33,34]. Superparamagnetic iron oxide nanoparticles (SPIONs) were found to induce selective toxicity only towards oral squamous cell carcinoma and not normal cells. Exposure to the superparamagnetic iron oxide nanoparticles (SPIONs) reduced the activity of succinate dehydrogenase in complex II of the mitochondria obtained from cancerous oral squamous [35] cells. A decrease in mitochondrial succinate dehydrogenase activity, in turn, increases the level of reactive oxygen species (ROS) and subsequent decline in mitochondrial membrane potential, the release of mitochondrial cytochrome complex, and mitochondrial swelling. The SPIONs also increased the lipid peroxidation level and caspase-3 activity in oral squamous carcinoma cells [36]. Therefore, the SPIONs can induce selective cytotoxicity towards the mitochondria of oral squamous cancer without significant effects on the normal mitochondria. On the other hand, zinc-oxide nanoparticles also showed increased cytotoxicity toward CAL-27 oral cancer cells by PINK1/ Parkin-mediated mitophagy [18]. Similarly, Li et al. found that stannic oxide nanoparticles can exert selective cytotoxic effects on oral cancer cells by inhibiting cell proliferation, migration, and invasion abilities and can also induce oxidative stress and apoptosis [37]. Although the underlying mechanisms are not fully understood, compatible with previous studies, our results suggest that GNSbs induced selective toxicity toward CAL-27 oral cancer cells.

Different from hydrophobic molecules, which can diffuse through the lipid bilayer membrane of cells, nanoparticles (NPs) require active uptake mechanisms to enter cells. When nanoparticles are present in biological fluids, biomolecules including proteins can form a “protein corona” by modifying the surfaces of the nanoparticles [38]. The composition of the protein corona (such as the type, amount, and conformation of the proteins) becomes the biological identity of the nanoparticles [39]. Therefore, the surface charge of nanoparticles has proven to be a major determinant of cellular internalization, with charge-based uptake highly dependent on cell type [40]. Previous studies on surface-dependent particle uptake showed that no simple rules have been identified so far. However, in general, cationic NPs appear to cause plasma membrane disruption to a greater extent and are ingested by nonphagocytic cells to a higher extent. Anionic NPs are better ingested and act more cytotoxically in phagocytic cells [41]. As CAL-27 oral cancer cells are epithelial cells and classified as non-professional phagocytic cells, our results suggested that CAL-27 cells showed a high level of GNSb uptake at 24 h. Because radiation treatment should be given accordingly after the administration of GNSbs for oral cancer cells to achieve optimal therapeutic effects, it is important to take note of the timing of the highest uptake of GNSbs by CAL-27 cells.

Single-strand breaks (SSBs), double-strand breaks (DSBs), and DNA base modifications are the most common types of DNA damage [42]. Among them, DSBs are the most detrimental. The failure to repair DSBs consequently leads to cell death. One of the possible mechanisms of radiosensitization by gold nanoparticles is the inhibition of DNA repair. Although the involvement of gold nanoparticles in DNA repair inhibition remained inconclusive [9], there were studies that showed that the number of γ-H2AX and 53BP1 foci increased in the presence of gold nanoparticles [43]. These findings suggested delayed DNA repair under the treatment of gold nanoparticles in cancer cells. Further evidence also indicated increased DNA damage in cells treated with irradiation with the addition of gold nanoparticles [44]. In our studies, although the mechanisms of decreased DNA DSBs in L929 cells after irradiation in the presence of GNSbs were not clear, the radiosensitization effect of GNSbs could be noted by the more prominent DNA DSBs in CAL-27 cells.

The effects of gold nanoparticles on cell cycle disruption vary and depend on the nanoparticles’ physicochemical properties and the types of cells [9]. There were some studies suggesting no significant influence of gold nanoparticles on cell cycle progression [44,45]. However, Mackey et al. reported that 30 nm nuclear-targeted gold nanoparticles (NLS-AuNPs) with 5-fluorouracil effectively increased the accumulation of squamous carcinoma cells in the S phase and caused the depletion of cells in the G2/M phase [46]. In contrast, other studies showed that glucose-capped gold nanoparticles caused the accumulation of cells in the G2/M phase [47]. Cell cycle distribution of a similar pattern was also reported with gold nanoparticles in breast, lung, ovarian, and melanoma cancer cells [48,49,50]. Evidence suggested that gold nanoparticles activate the cyclin-dependent kinases, leading to cell cycle acceleration in the G0/G1 phase and accumulation in the G2/M phase. G2/M arrest was accompanied by decreased expression of p53 and cyclin A and increased expression of cyclin B1 and cyclin E [47]. In this study, our results showed that GNSbs with irradiation induced significantly more CAL-27 remaining in the G2/M phase than L929 cells after 4 h of treatment (Figure 7A,B). Moreover, the cell cycle histogram at 48 h after treatment showed that GNSbs with irradiation induced more G2/M phase arrest in CAL-27 than L929 cells (Figure 7C). Although further investigations are necessary to clarify the mechanisms of nanoparticles on perturbations in the cell cycle, these important findings provide insights into strategies for designing effective serial radiation therapy.

## 5. Conclusions

In this study, new gold nano-sesame-beads (GNSbs) of mesoporous silica nanobeads with pores filled with gold nanorods were successfully developed. The GNSbs showed selective toxicity and higher cellular uptake in oral squamous carcinoma cells. The combination of GNSbs and radiotherapy further increased the cytotoxicity against squamous carcinoma cells. These sensitization effects of GNSbs to ionic radiation may have been related to intracellular ROS production, impaired DNA DSB repair, and cell cycle accumulation in the G2/M phase. In the animal studies using an orthotopic model, GNSbs showed encouraging radiosensitization effects and provided better control over average tumor size than irradiation alone. However, more animal studies should be conducted in the future to provide statistically significant evidence. However, GNSbs still open up a potential avenue for treating oral squamous carcinoma due to their specific cellar uptake and radiosensitization properties. Further studies on the detailed mechanisms of GNSbs are essential for a greater understanding and in the design of future clinical treatment strategies.

## Data Availability

Not applicable.
